# The role of astrocyte‐mediated plasticity in neural circuit development and function

**DOI:** 10.1186/s13064-020-00151-9

**Published:** 2021-01-07

**Authors:** Nelson A. Perez-Catalan, Chris Q. Doe, Sarah D. Ackerman

**Affiliations:** 1 Institute of Neuroscience, Howard Hughes Medical Institute, University of Oregon, Eugene, OR USA; 2 Kennedy Center, Department of Pediatrics, The University of Chicago, Chicago, IL USA

**Keywords:** Astrocyte, Synapses, Circuits, Hebbian plasticity, Homeostatic plasticity, Gap junction

## Abstract

Neuronal networks are capable of undergoing rapid structural and functional changes called plasticity, which are essential for shaping circuit function during nervous system development. These changes range from short-term modifications on the order of milliseconds, to long-term rearrangement of neural architecture that could last for the lifetime of the organism. Neural plasticity is most prominent during development, yet also plays a critical role during memory formation, behavior, and disease. Therefore, it is essential to define and characterize the mechanisms underlying the onset, duration, and form of plasticity. Astrocytes, the most numerous glial cell type in the human nervous system, are integral elements of synapses and are components of a glial network that can coordinate neural activity at a circuit-wide level. Moreover, their arrival to the CNS during late embryogenesis correlates to the onset of sensory-evoked activity, making them an interesting target for circuit plasticity studies. Technological advancements in the last decade have uncovered astrocytes as prominent regulators of circuit assembly and function. Here, we provide a brief historical perspective on our understanding of astrocytes in the nervous system, and review the latest advances on the role of astroglia in regulating circuit plasticity and function during nervous system development and homeostasis.

## Background

Nervous system assembly requires precise coordination between the formation of millions of synapses and integration of these synapses into functional circuits. Failure to do so leads to significant life-altering neurodevelopmental and neurodegenerative disorders [1–5]. Between 2006 and 2008, approximately 15% of children aged three to seventeen were affected by neurological disorders in the United States alone [6]; thus, understanding the mechanisms that regulate proper neural development will have a direct impact on human health.

**Fig. 1:**
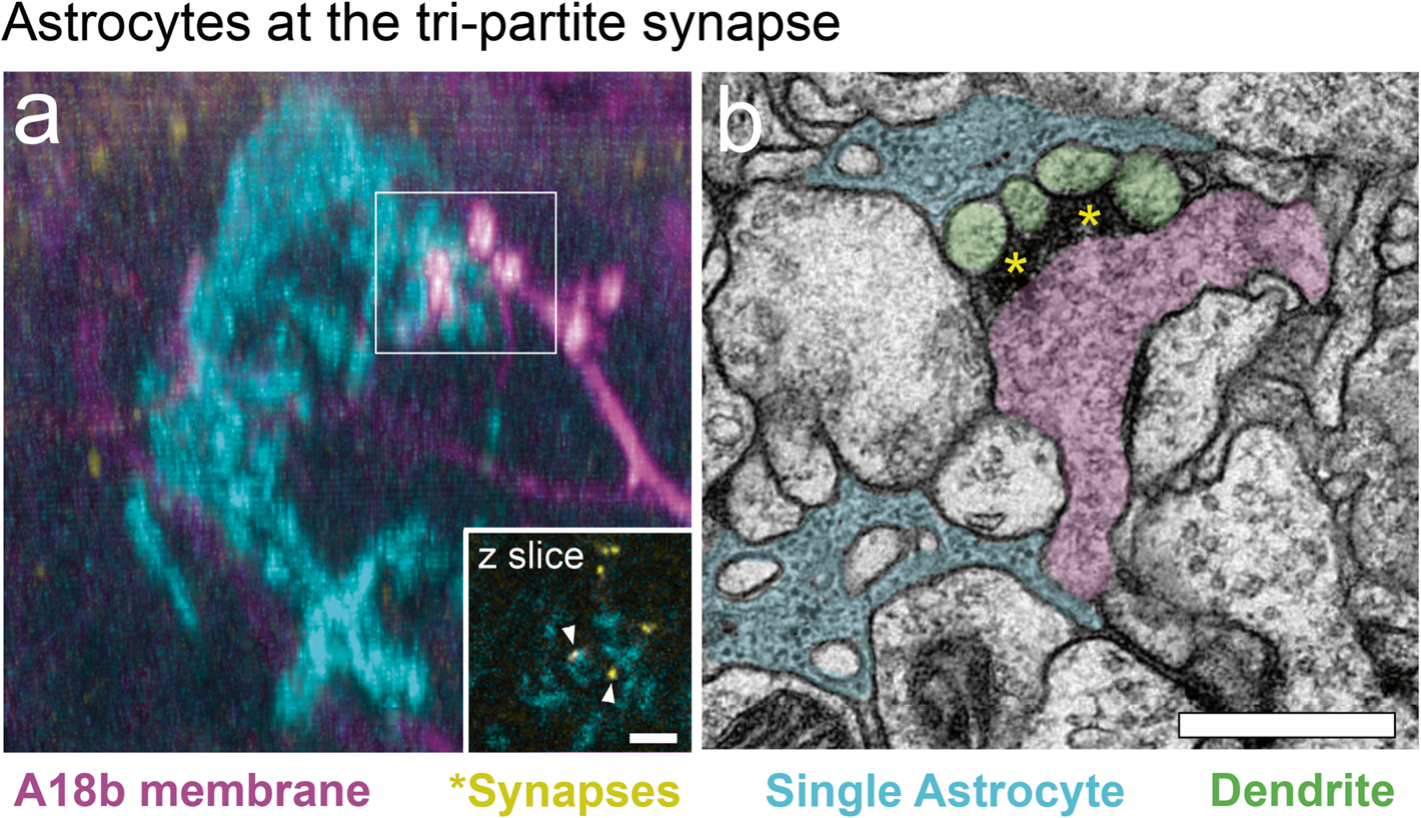
Astrocytes locally support neuronal synapses. **a** Light microscopy image of single astrocyte (cyan) contacting the pre-synaptic membrane of the *Drosophila* A18b neuron (magenta), with pre-synapses highlighted in the inset (yellow). First instar larva. Genotype: A18b (*94E10-lexA; 8xlexAop-2xBrp-short::cherry; lexAop-myr::GFP*), astrocyte (*25h07-gal4; hs-FLPG5;; 10xUAS(FRT.stop)myr::smGdP-HA, 10xUAS(FRT.stop)myr::smGdP-V5, 10xUAS-(FRT.stop)myr::smGdP-FLAG*). Scale bar, 200 nm. **b** TEM image showing a single astrocyte (cyan) contacting the pre-synaptic membrane of the A18b neuron (magenta), and the post-synaptic membrane of an A27a neuron (green) with synapses highlighted (yellow asterisks). Genotype: wild type. First instar larva. Scale bar, 500 nm

Glial cells help coordinate synapse formation and circuit assembly. Additionally, they monitor, instruct, and support neuronal activity in mature circuits [6, 7]. Astrocytes, the most abundant subtype of glial cells in the central nervous system (CNS), are classically known for their roles in neurovascular coupling and metabolic support of neurons during homeostasis [8]. In the mature nervous system, astrocytes directly contact the neuronal soma, dendrites, spines, and presynaptic terminals (Fig. 1) [9], thus they are uniquely poised to regulate neuronal function.

### Astrocyte heterogeneity in the nervous system

Astrocytes have been described as a homogenous population of cells since their discovery [10], yet, a growing body of evidence now suggests that astrocytes are highly diverse in their morphology, gene expression profiles, and functionality [11–16]. For example, in the developing vertebrate spinal cord, differential expression of morphogens from the dorsal and ventral poles generates neurons belonging to eleven distinct functional domains organized along the dorsal-ventral axis [17]. Astrocytes tile throughout the vertebrate spinal cord, and interestingly, spinal cord astrocytes also show domain-specific expression profiles, leading to the hypothesis that domain-specific astrocytes represent subclasses. Indeed, a recent report found that depletion or mutation of astrocytes from the pMN domain of the mouse spinal cord disrupted sensorimotor circuit formation and maintenance, and that astrocytes from neighboring domains do not repopulate the region to compensate [12, 13]. More recently, in situ single-cell gene expression analyses of cortical astrocytes found laminar organization of astrocyte transcriptomes, as well as markers for superficial, mid and deep astroglial populations in adult mouse and human cortex [18]. Disruption of neuronal differentiation in murine cortical layers L2-4 resulted in aberrant astrocyte organization in the superficial layers. Moreover, inversion of neuronal layers in the cortex at postnatal day 14 (P14) resulted in similarly upturned astrocytic marker expression. Together, these data demonstrate that astrocytes show region-specific expression and function [19]. It remains to be tested whether these changes are astrocyte-intrinsic, or whether the neuronal microenvironment resolves local astroglial identity.

### Astrocyte expansion coincides with synapse development

Recent data suggest that astrocytes are also critical regulators of nervous system development [8, 20]. Indeed, studies from multiple model systems suggest that the expansion of astrocytic membrane domains occurs in tandem with the birth and refinement of synapses within individual circuits, including visual processing, attention, memory, and motor control pathways [21–28]. Astrocytes extend fine processes to establish non-overlapping territories after the first postnatal week in the developing mouse cortex, coincident with synaptogenesis [29]. The timing of astrocyte migration and expansion in the developing spinal cord occurs earlier in rodents, ranging from late prenatal stages to postnatal day seven [30]. Similarly, astrocytes extend processes into the *Drosophila* ventral nerve cord (analogous to the vertebrate spinal cord) during the final stage of embryogenesis [31], and by 6 days post-fertilization in the developing zebrafish spinal cord [32]. In humans, astrocytes are born in late fetal stages [33]. Although studies of human astrocyte development are challenging and a precise timecourse of human astrocyte-synapse association has yet to be done [34, 35] a single cortical human astrocyte can extend processes from the soma that gradually ensheath upwards of two million individual synapses [36]. It is noteworthy to mention that human astrocytes are larger, more structurally complex, and more diverse than astrocytes in any other chordates assessed to date [14, 36–39]. In each case, the expansion of astrocytic membranes into the neuropil occurs alongside synaptogenesis. Together, these studies intimated that astrocyte-derived cues could influence synapse development, and vice versa.

## Astrocyte regulation of synapse number

Given the relatively late timecourse of astrogenesis during nervous system development, astrocytes are not present to regulate embryonic waves of neurogenesis and axon outgrowth. Though outside the scope of this review, note that astrocytes do form part of the neurogenic niche that regulates adult neurogenesis (reviewed in [40]), and that reactive astrocytes regulate axon regrowth and recovery after nervous system injury [41]. During early postnatal development, astrocytes elaborate their fine processes concurrent with synaptogenesis. The timing of astrocyte arrival in the CNS, and their strategic positioning of peri-synaptic processes, make astrocytes an attractive candidate to promote assembly of neurons into neural circuits. We now appreciate that these roles include, but are not limited to, structural and functional synaptogenesis [21, 25, 26, 42], synapse pruning [43, 44], and synapse maintenance [13].

### Astrocytes in synaptogenesis

A role for astrocytes in synaptogenesis was first defined in the lab of Dr. Ben Barres by taking advantage of mouse retinal ganglion cell (RGC) culturing systems. These pioneering studies demonstrated that addition of astrocytes to neuronal cultures was sufficient to promote synapse formation and spontaneous activity of RGC neurons, which are largely inactive in the absence of glial support [28, 45, 46]. A similar role for astrocytes in promoting synaptogenesis using rat RGC microcultures was described shortly thereafter [47], and more recently in cultured human cerebral cortical spheroids [48]. Together, these data provide direct cross-species evidence that astrocytes are able to directly promote circuit development and function *in vitro*. Genetic access to astrocytes during circuit assembly was not available until recently [49–53], yet these advances have rapidly expanded our understanding of the contribution of astrocytes to circuit development. Advances on invertebrate and vertebrate *in vivo* animal models demonstrate that astrocytes are regulators of neural circuit assembly in *C. elegans* [54, 55]; *Drosophila* [56, 57]; feline [58]; *Xenopus* [59]; rodent [8, 25, 28, 47]; and human [60, 61].

Over the last decade, we have greatly expanded our understanding of the molecular mechanisms by which astrocytes regulate synaptogenesis. Astrocyte-derived (secreted and membrane bound) synaptogenic and anti-synaptogenic cues dynamically interact to finely tune synapse number during neural circuit assembly [8, 62]. As these pathways have been extensively covered elsewhere [8, 63], here we focus specifically on Hevin and SPARC, which are essential for the generation of functional synapses during mammalian nervous system development, and also regulate synapse plasticity (discussed below) [26]. These proteins are of additional interest given that the upregulation of their expression profiles has been linked to neurodevelopmental disorders [64] and reactive astrogliosis in adults [65–67]. The matricellular protein Hevin is secreted by astrocytes localized to excitatory CNS synapses throughout the organism’s life, and peaks in its expression during synaptogenesis and following CNS injury [14, 67–69]. Extensive studies of retinocollicular and thalamocortical synapse development have demonstrated that Hevin is required for the formation and maturation of glutamatergic synapses [26, 27, 70]. In the latter case, Hevin refines thalamic presynaptic inputs onto cortical dendrites by bridging pre-synaptic Neurexin-1α to dendritic Neuroligin-1B, and loss of Hevin causes a reduction of mature glutamatergic synapses [27]. Astrocytes also produce SPARC (Secreted Protein Acidic and Rich in Cysteine), which acts as a competitive inhibitor to antagonize Hevin-induced synaptogenesis. According, while Hevin null mice show decreased numbers of excitatory synapses in the superior colliculus, SPARC KO mice show enhanced synaptogenesis in the same brain region at P14 [26]. Interestingly, SPARC does not inhibit excitatory synaptogenesis induced by astrocyte-derived thrombospondins, but is a specific antagonist of Hevin. Because these proteins are not known to physically interact, it remains to be seen how Hevin and SPARC function together to tune synapse number *in vivo* [26]. More recently, a novel *in vivo* enzymatic assay defined a proteome for extracellular astrocyte-neuron junctions in the primary visual cortex (V1 cortex) and found that astrocytic Neuronal Cell Adhesion Molecule (NRCAM) binds to NRCAM-gephyrin complexes on postsynaptic neurons to induce the formation and function of inhibitory GABAergic synapses, with only minor effects on excitatory synapses [71]. Together, these results identify a direct role for astrocytes in the control of excitatory and inhibitory synapse assembly and maturation *in vivo*, while also displaying the heterogeneity of astroglial cues depending on the synapse subtype.

### Astrocytes in synapse pruning

**Fig. 2:**
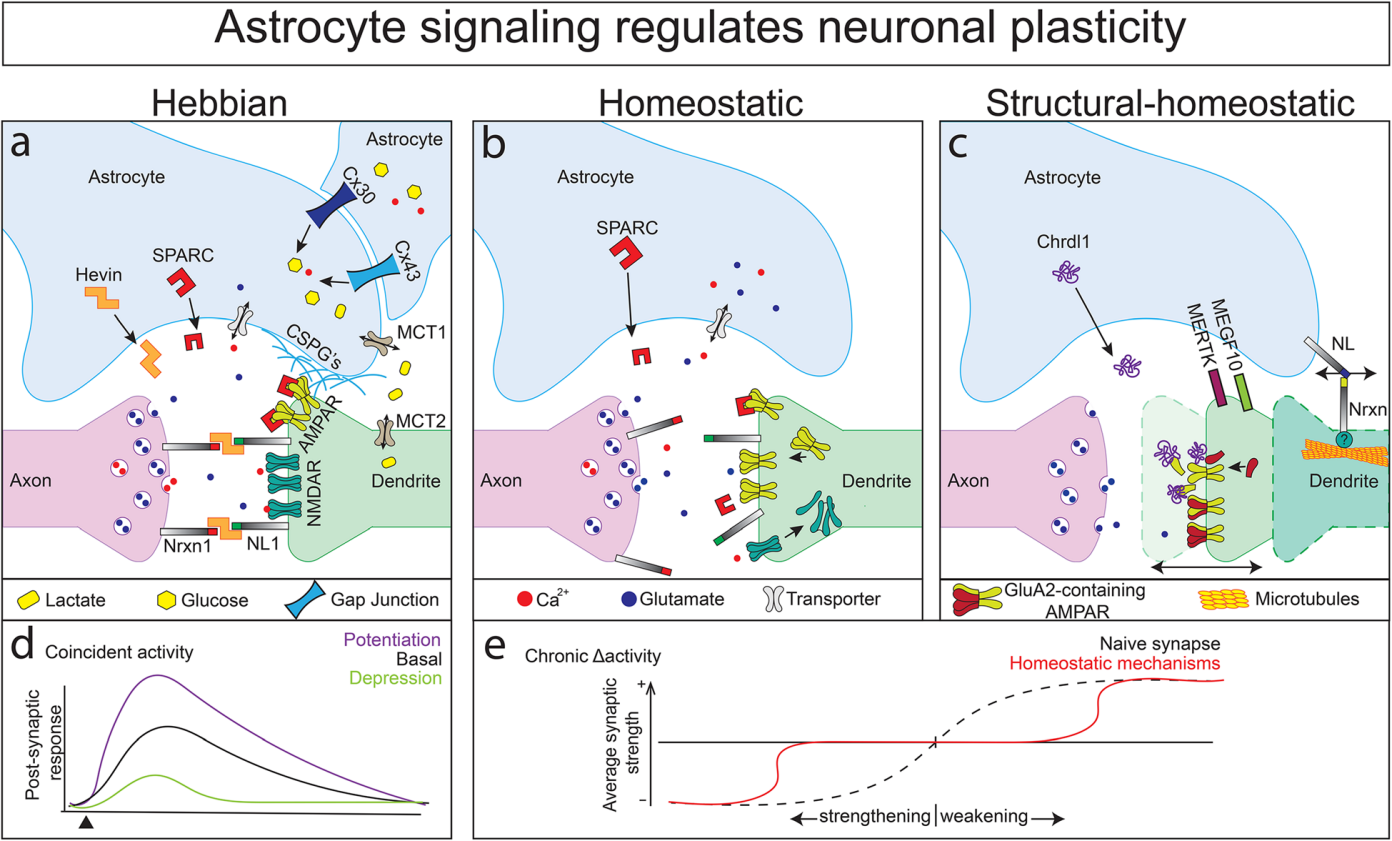
Select mechanisms for astrocyte-induced plasticity. **a **Hebbian plasticity. Recruitment of NMDA receptors is mediated by astrocyte-derived Hevin and the cell adhesion molecules Neuroligin-1 (NL1) and Neurexin-1 (Nrxn1) during the ocular dominance plasticity critical period. Astrocyte chondroitin sulfate proteoglycans (CSPGs) and SPARC stabilize AMPA postsynaptic receptors. Astrocyte gap junction proteins Connexins 30 and 43 regulate metabolite transport through monocarboxylate transporters (MCT1/2) between astrocytes and neurons in an activity-dependent manner to facilitate plasticity. **b **Homeostatic plasticity. Astrocyte-derived SPARC limits aggregation of AMPA receptors to facilitate synaptic scaling in response to chronic silencing. Additionally, receptors and transporters located in the astrocytic membrane monitor neuronal Ca^2+^ transients and release of neurotransmitters, resulting in gliotransmitter release. **c **Structural-homeostatic plasticity. Astrocyte-secreted Chrdl1 restricts neuronal plasticity by directly switching postsynaptic neurotransmitter receptor identity. Astrocyte-derived Neuroligin (NL) binds dendritic Neurexin (Nrxn) to mediate the closure of critical periods by stabilizing dendrite microtubule populations. Synapse elimination is driven by neuronal activity, and is regulated by astroglial MERTK and MEGF10. **d** Repeated excitatory postsynaptic potentials evoke more robust synaptic activity in potentiated circuits over time. Conversely, synapses targeted by long term depression display lower levels of excitability following stimulation. **e **Homeostatic mechanisms decrease the difference between synaptic input and output by bidirectionally adjusting the probability of transmitting an action potential postsynaptically

Overproduction of synapses and their subsequent experience-dependent elimination is critical for refinement of neuronal circuits during development [72]. This is especially well-characterized during ocular dominance plasticity (discussed further below), and during *Drosophila* circuit rewiring in metamorphosis and regeneration [43, 59]. In 2013, Chung et al., demonstrated that astrocytes and microglia participate in synapse elimination via two activity-dependent phagocytic receptors, MEGF10 and MERTK, which trigger engulfment of excitatory and inhibitory synapses in the developing mouse visual system (Fig. 2c). Loss of MEGF10 in mouse results in a failure to refine retinogeniculate connections in the developing visual system, resulting in ectopic synapses with reduced functionality [43]. Similarly, the *Drosophila* homolog of MEGF10, Draper, is necessary for clearance of axons during injury and during circuit remodeling [44, 73, 74]. A feature of the adult CNS is its ability to engage in activity-dependent synaptic plasticity during learning and memory [75]. Strikingly, MEGF10 and MERTK-dependent synaptic engulfment by astrocytes continues through adulthood in both murine and human cortical layers, which may contribute to learning, memory, and disease [43, 76, 77]. A recent report in *Drosophila* discovered that during a critical period of brain development in young adults, the extracellular domain of the amyloid precursor protein-like (APPL, homologous to human APP) regulates glial expression of Draper and clearance of neuronal debris after injury [78]. It will be interesting to test whether APPL/APP also regulates developmental pruning of synapses. Thus, the number of synapses on a neuron is not exclusively an intrinsic property but is heavily regulated by glial signals.

## Astrocytes tune synapse function and synaptic plasticity

The establishment of functional neuronal circuits does not only depend on early synaptogenic and pruning processes. To achieve precise CNS wiring, the developing nervous system must be able to adapt to the onset of neural activity, which can induce extensive, activity-dependent functional and structural remodeling of mature synapses [79]. Also known as plasticity, these restructuring events are usually driven by the arrival of environmental stimuli via sensory afferents [80, 81]. The progression from immature to functionally effective neuronal circuits that drive robust behavior is dependent on careful regulation of short- and long-term remodeling events. These events are strongly enriched during developmental windows called critical periods [80, 82]. If changes in synaptic strength are not carefully regulated, the activity passing through a given neuronal circuit could increase or decrease unchecked, resulting in abnormal activity patterns, the loss of sensitivity for synaptic partners, or excitotoxicity [79, 83, 84].

Functional and structural modifications to synapse and circuit function are generally categorized as either *Hebbian* or *homeostatic* plasticity (Fig. 2a-c). During Hebbian plasticity, coincident activity at pre- and post-synaptic sites causes modifications that alter synaptic efficacy through a positive feedback loop (Fig. 2d). The most widely studied form of Hebbian plasticity is long-term potentiation (LTP), which underlies long-term memory [79, 85–87]. Hebbian plasticity usually occurs at single synapse scale rather than circuit-wide scale, where an increase in presynaptic firing increases the probability of a further increase in postsynaptic gain [79, 88]. Conversely, homeostatic plasticity is a negative feedback mechanism that is activated in response to chronic changes to activity and serves to prevent runaway excitation/inhibition in response to Hebbian plasticity (Fig. 2e). Although homeostatic plasticity can function on individual synapses [89, 89], it also functions on the scale of whole neurites, neurons, and even to balance levels of activity through an entire circuit via functional and structural remodeling [88, 90–93]. The changes that arise from these dynamic remodeling events can have profound effects on circuit function, behavior, and human health [82], yet the developmental mechanisms that promote or restrict plasticity are not yet fully understood at the cellular or molecular level.

### Astrocytes regulate hebbian plasticity, one synapse at a time

Following the discovery of Hebbian plasticity over half a century ago [85], many different forms of remodeling have been identified, including both local (synaptic) and circuit-wide [79, 80, 86]. However, studies of neurons alone have failed to reveal how circuit plasticity is established and circuit balance is maintained. As mentioned above, RGCs cultured in the presence of astrocytes show elevated neuronal activity [46]. Recent advancements in microscopy and genetic strategies for monitoring glial cell populations have led to a new awareness for how astrocytic networks are strategically arranged to support and modify synaptic activity [47, 94, 95].

In the mammalian CNS, glutamate triggers ion flow through N-methyl-D-aspartate receptors (NMDARs) on postsynaptic membranes to power excitatory neurotransmission [96]. Repeated stimulation of sensory and learning pathways (such as those in the hippocampus) can enhance recruitment of NMDARs to the postsynaptic terminal, thereby increasing the efficacy of long-term synaptic transmission (e.g. LTP) [97, 98]. In addition, the concentration of α-amino-3-hydroxy-5-methyl-4-isoxazolepropionic receptors (AMPARs) on postsynaptic membranes can alter short-term synaptic plasticity [99]. As aforementioned, astrocytes secrete the matricellular protein Hevin, which increases the number and size of excitatory synapses in RGC cultures and *in vivo* during development of the visual system [26, 27, 70]. Recent studies revealed an additional role for Hevin in neuronal plasticity. During ocular dominance plasticity (ODP), monocular deprivation weakens synapses downstream of the closed eye, while cortical connections downstream of the open eye are coincidently strengthened [58, 100–102]. This process is dependent on the differential recruitment of post-synaptic NMDARs [101, 103–105]. Astrocyte-derived Hevin organizes presynaptic Neurexins and postsynaptic Neuroligins (binding partners), thereby aligning the pre-synaptic neurotransmitter release machinery with post-synaptic NMDARs during the ODP critical period (Fig. 2a) [27, 106]. Accordingly, *Hevin* null mice exhibit reduced ODP, which can be rescued upon viral delivery of Hevin to astrocytes [27]. Interestingly, though Hevin and SPARC have opposing functions in synaptogenesis, synaptic plasticity (LTP) is also reduced in the hippocampus of SPARC null mice. Reduced LTP in SPARC null animals is not the result of decreased NMDAR localization, but rather increased levels of AMPARs on postsynaptic membranes and enhanced baseline activity of these synapses [107]. These data suggest that during developmental plasticity, Hevin and SPARC function together to fine-tune the ratio of NMDARs to AMPARs to facilitate LTP. Overall, these data demonstrate that astrocytes modulate synaptic strength by regulating post- and presynaptic receptor composition.

### Astrocyte tiling and synaptic transmission

Bidirectional communication between astrocytes and synaptic terminals is critical for the establishment and maintenance of neuronal transmission [8, 106]. A property of astrocytes that expands the complexity of these interactions is the capacity of a single astrocyte to associate with millions of synapses within an expanded glial network. This network is generated by gap junctions (GJs) that allow neighboring astrocytes to tile with one another, yet maintain non-overlapping territories [108–110]. GJs are intercellular channels that integrate astrocytes into functional syncytia, which facilitate signaling and transport of metabolites between neuronal and non-neuronal tissues [111, 112]. The GJ proteins connexin 30 (Cx30) and connexin 43 (Cx43) are highly expressed in astrocytes [69, 113], and act to shuttle glucose and its metabolites (lactate) from astrocytes to neurons in an activity-dependent manner (Fig. 2a) [111, 114, 115]. In the absence of astroglial Cx30 and Cx43, astrocyte-dependent glutamate recycling and potassium buffering at the synapse is impaired. This results in enhanced excitability at hippocampal CA1 Schaffer collateral synapses due to increased levels of AMPARs [94]. Interestingly, analysis of synapse numbers on CA1 neurons of *Cx30*^*−/−*^*Cx43*^*−/−*^ mice revealed that while the overall number of synapses remains unchanged in the absence of GJs, the pool of silent synapses was significantly decreased. This led to defects in Hebbian plasticity, including suppressed LTP and increased occurrences of long-term depression. Disruptions to astrocyte GJ proteins have been linked to impairments in LTP during memory allocation and stress in the mammalian hippocampus [115–117]; thus, GJ-dependent astrocyte communication is critical for developmental learning and behavior.

### Astrocyte‐derived extracellular matrix modifies synaptic plasticity

Finally, astrocytes may influence synaptic efficacy through modulation of the extracellular environment [118]. Chondroitin sulfate proteoglycans (CSPGs) are glycosylated, extracellular matrix proteins secreted by both neurons and glia that form integral components of perineuronal nets (PNNs). PNNs are lattice-like aggregates of CSPGs surrounding neuronal processes [119]. PNNs emerge in concert with the arrival of astrocytes during late postnatal development, corresponding with the closure of critical periods of experience-dependent plasticity [82, 120]. CSPGs act as lateral diffusion barriers for AMPARs, and can therefore facilitate post-synaptic receptor composition to modulate short-term synaptic plasticity (Fig. 2a) [121]. It was recently shown that removal of PNNs from the mouse deep cerebellar nuclei increased synaptic plasticity and improved active learning during eyeblink conditioning, a form of motor learning. Conversely, loss of PNNs inhibited the formation of eyeblink-associative memories [122]. Although astrocytes express a variety of CSPGs during nervous system development [14, 69] and following injury [123–125], the relative contribution of astrocyte to neuron-derived ECM for circuit development and plasticity remains poorly defined [126, 127]. An important open question is whether astrocytic CSPGs modulate AMPAR aggregation *in vivo* during circuit assembly. In addition, it remains unknown whether astrocyte-derived and neuronal-derived CSPGs trigger different signaling cascades. This is especially critical to determine because altered CSPG signaling is linked to poor outcomes in injury and disease [123–125, 128–131].

## Astrocytes are key regulators of homeostatic plasticity, from synapses to circuits

Homeostatic plasticity arises in response to prolonged changes in network activity, shifting the balance away from extreme excitation (E) or inhibition (I) to maintain E/I balance. Thus, a neuron can preserve its ability to respond to activity via Hebbian plasticity by maintaining a functional state of excitability [132]. This form of plasticity was first theorized to exist as a “normalizing” force in mathematical models of circuit function [133–135], as the technology to detect, characterize, and confirm homeostatic plasticity was not available until much later [136–141]. Homeostatic plasticity has the capacity to modify multiple substrates within neural circuits. Synaptic scaling is a homeostatic mechanism that alters the strength of individual synapses, which can modify the activity and function of neurotransmission for a single neuron [132, 142]. Synaptic scaling is a modification to the number of AMPAR at the post-synaptic terminal, which can reduce synaptic strength and the probability of a postsynaptic potential. Modifications to neurotransmission through synaptic scaling can take place either by removing AMPARs locally at the terminal [143], or globally, by affecting the rate of transcription of AMPARs within a circuit [144]. Although several neuronal mechanisms for homeostatic plasticity have been defined [141], a looming question is to determine how this form of plasticity is regulated at the circuit level. The juxtaposition of synapses and perisynaptic processes of astrocytes (Fig. 1) makes them a great target to study regulation of homeostatic plasticity [88, 145].

We now understand that astrocytes secrete a number of proteins that modulate homeostatic plasticity at the synaptic and circuit level. A well-documented example is SPARC [146, 147]. As noted above, analysis of cultured hippocampal slices from astrocyte-specific SPARC knock-out mice revealed increased numbers of postsynaptic GluR2 receptor subunits, which caused an ectopic accumulation of postsynaptic surface AMPARs and impaired LTP following high frequency stimulation [107]. Accordingly, loss of SPARC inhibits synaptic scaling following activity deprivation by TTX (Fig. 2c) [107]. These data demonstrate that astrocytes can co-opt the same signaling pathways to regulate both Hebbian and homeostatic plasticity during circuit assembly.

During critical periods, activity across circuits can also alter neuronal architecture, ranging from retraction/extension of individual synaptic elements to modifications to dendrites and axons. This form of plasticity, also known as homeostatic structural plasticity, can drastically affect the probability of forming synapses between neighboring neurons by physically increasing or decreasing membrane space [88, 90, 92, 93, 148, 149]. Recent work has defined important roles for astrocytes in homeostatic structural plasticity [42, 93, 150]. In the CNS, astrocyte-secreted Chordin like-1 (Chrdl1) was shown to regulate the switch from AMPAR to GluA2-containing synapses in the developing mouse cortex [42]. Moreover, in a monocular enucleation model of ocular dominance homeostatic plasticity (reviewed in [151]), Chrdl1 knock-out resulted in ectopic synaptic remodeling events, suggesting that astrocytes use Chrdl1 to restrict neuronal plasticity (Fig. 2c). Excitingly, a recent report demonstrated that astrocytes close a critical period of motor dendrite remodeling in the *Drosophila* CNS via astrocyte Neuroligin to motor neuron Neurexin signaling (Fig. 2c) [93]. Importantly, this study pinpoints astrocytes as a putative target in critical period-dependent neurodevelopmental disorders [152].

## Astrocytes shape neuronal signaling

**Fig. 3:**
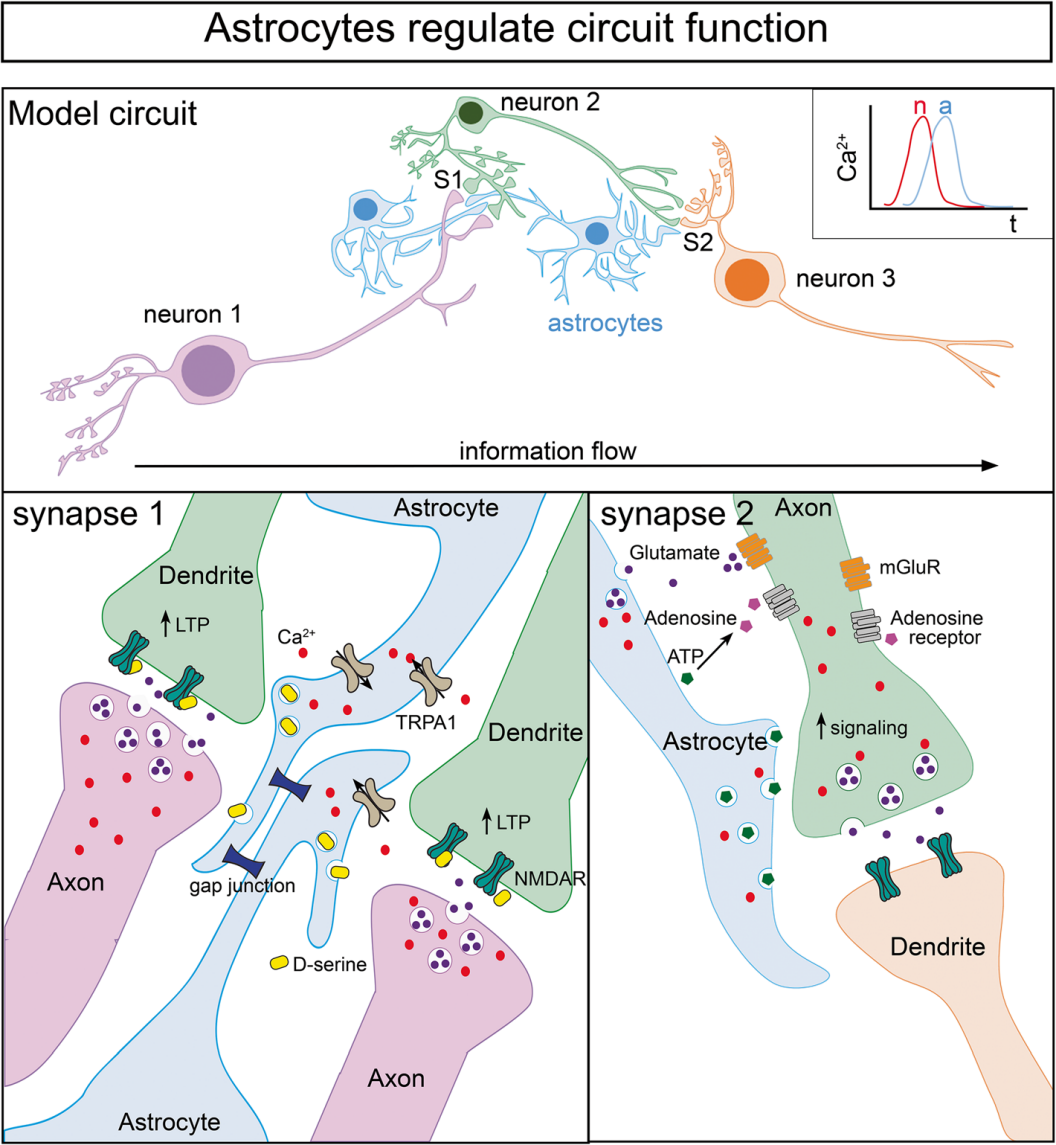
Select mechanisms for astrocyte-modulated neuronal signaling. Top: Simple model circuit showing three linearly connected neurons (n) and associated astrocytes (a). at two synaptic (S) connections. Synapse 1: Influx of calcium into gap junction-coupled astrocytes via TRPA1 channels occurs in response to local neuronal activity. Elevation of astrocytic calcium causes astrocyte activation and release of gliotransmitters including D-serine, which induces NMDAR-dependent LTP. Synapse 2: Elevation of astrocytic calcium can also drive release of the gliotransmitters ATP and glutamate, which can stimulate pre-synaptic adenosine and metabotrophic glutamate (mGluR) receptors, respectively, to promote signaling of downstream neurons and drive the flow of information through the circuit

Although astrocytes themselves are not electrically excitable, astrocytes measure local synaptic activity via metabotropic and ionotropic-sensing receptors [153–155]. Interestingly, astrocytes display Ca^2+^ transients that mirror neuronal activity [156–160]. In *Drosophila*, astrocyte Ca^2+^ signaling in the ventral nerve cord occurs in parallel with motor waves [161]. Similarly, co-imaging of Ca^2+^ transients in astrocytes and neurons following mouse whisker stimulation demonstrated that neuronal and glial calcium waves operate in synchrony during sensory tasks [162, 163]. Voluntary limb movements have also been shown to cause Ca^2+^ elevations of motor cortex astrocytes in awake, moving mice, suggesting efferent activity is tightly coupled to astrocyte activation [164]. It remains unclear whether astrocytic Ca^2+^ waves originate within the gap junction-coupled astroglial network, or represent the product of the linear summation of neighboring neuronal activity [165–169]. Nevertheless, we now understand that activity-dependent calcium elevation in astrocytes can induce release of small molecules including glutamate, ATP, and D-serine [170–173] in a calcium- and SNARE protein-dependent mechanism [174]. In turn, these “gliotransmitters” modify synaptic transmission and short-term plasticity (Fig. 3) [155, 161, 175–179]. For example, induction of calcium transients within astrocytes by direct manipulation or via inositol tris-phosphate-dependent signaling has been shown to depress or enhance synaptic transmission [154, 175, 180]. Recently, Ma et al. (2016) demonstrated that a *Drosophila* TRPA1 calcium channel (Water witch) is expressed in astrocytes and facilitates the accumulation of calcium in response to local neuronal activity. Astrocytic calcium can in turn modulate downstream dopaminergic neuron activity and locomotor behavior *in vivo* [161]. Indeed, it is now apparent that astrocytes are essential for rhythmic locomotor behaviors [93, 181–183]. In mouse, the sensory TRPA1 Ca^2+^ channel similarly maintains basal astrocytic calcium levels to facilitate constitutive D-serine release at the synapse. Loss of TRPA1 impairs NMDA-dependent LTP at Schaffer collateral to CA1 pyramidal neuron synapses, demonstrating that fine astrocytic processes tune synaptic plasticity in an activity-dependent manner [184]. There is mounting evidence that astrocyte calcium signaling arises to modulate sensory-evoked neuronal activity. Well documented examples include somatosensory stimulations that trigger Ca^2+^ elevations in astrocytes which amplify stimulus-evoked cortical plasticity via noradrenaline and acetylcholine [185–188]. More recently, Lines et al. (2020) correlated the modulation of somatosensory afferents to astrocyte Ca^2+^ waves, and showed that astrocyte activation dampens sensory-evoked neuronal activity in S1 (primary somatosensory cortex) [160]; thus, placing bidirectional astrocyte-neuron communication at the center of sensory information processing in the mammalian cortex. This opens an exciting line of future work, where astrocytic activation could be manipulated to modulate neuronal activity during critical periods of circuit development and disease.

Finally, astrocyte networks may contribute to circuit plasticity during memory allocation and goal-directed behaviors. Ectopic activation of astrocytes is sufficient to induce de novo NMDA-dependent LTP in CA3-CA1 pyramidal neurons [189, 190]. Interestingly, the authors also found that while direct neuronal activation impaired memory formation, delayed activation through astrocytes strongly enhanced memory allocation [190], suggesting that indirect signaling through astrocytes may be necessary to gate LTP. Indeed, a recent report suggests that gliotransmission by astrocytes recruits metabotropic glutamate receptors to the presynaptic terminal during spike timing-dependent plasticity, a process that shifts developing hippocampal synapses from long term depression (LTD) to LTP [191, 192] (Fig. 3). Extensive work in mouse models have also defined astrocytes as key regulators of inhibition. As aforementioned, astrocyte-derived NRCAM influences inhibitory synapse development and function in the developing visual cortex. Similarly, astrocytic activation in the limbic system can drive depression of excitatory synapses and enhancement of inhibitory synapses in the central amygdala [179]. In the developing somatosensory cortex, astrocytic signaling mediates spike-timing-dependent LTD [192, 193]; and in the developing prefrontal cortex, astrocytic GABA_B_ receptors monitor local concentrations of GABA and in turn, regulate low gamma oscillations (see more below) and goal-directed behaviors [194]. Thus, bidirectional signaling between neurons and astrocytes ensures proper E/I balance in multiple brain regions to shapes the flow of information through neural circuits and facilitate neuronal plasticity that is essential for learning, memory, and goal-directed behaviors.

## Astrocytes and circuit pattern generation

It is evident that astrocytes tune synaptic and circuit architecture during sensory-dependent plasticity. Yet, plasticity also occurs even during “idle” periods with little environmental input [195–197]. Since the discovery of electroencephalography [198], scientists have identified several rhythmic voltage fluctuations in the brain, from individual neurons to whole neuronal networks [199]. These oscillations emerge in all brain regions, and their patterns underlie the basis for internal day/night cycles, sensory representation, and short term memory [200, 201]. Astrocytes are capable of modulating neuronal rhythms by mediating ion homeostasis at the synapse [194, 202–206]. Interestingly, astrocyte-dependent ion homeostasis seems to be critical for oscillatory behaviors such as sleep [207, 208]. Indeed, a suite of papers describing the role of astrocytes in sleep in fly and mouse were published in the last six months alone. In brief, astrocytes exhibit calcium waves that follow natural circadian rhythms- they are highest during wake phases and lowest during sleep [207, 209]. Interestingly, astrocytes accumulate high levels of calcium during wake cycles in order to encode sleep need [209–211], similar to astrocytic calcium encoding futility-induced passivity in zebrafish [178]; enhancing astrocytic calcium caused perpetual sleep in *Drosophila* [210] and reducing astrocytic calcium is necessary for slow-wave sleep in mouse [207]. Finally, this transition in behavioral state is dependent on the local concentration of neurotransmitters (dopamine, serotonin, and endocannabinoids) sensed by astrocytic receptors [212, 213]. It will be interesting to test whether high levels of neuronal activity during the day also drive sleep recovery (e.g. napping) in an astrocyte-dependent manner. Additionally, as sleeping behaviors can change dramatically over the course of organismal development (e.g. neonate versus adult humans) or with the seasons (e.g. torpor), it will be important to determine the degree to which astrocytes contribute to sleeping behaviors outside of the standard diurnal clock. Thus, astrocytes coordinate both developmental and homeostatic circuit activity from the scale of individual synapses to circuits.

## Concluding remarks

Synaptic plasticity ensures the correct assembly and tuning of millions of synapses during nervous system development [82, 86]. From their perisynaptic location, astroglia have been shown to organize pre- and postsynaptic elements that modify Hebbian mechanisms of plasticity [27, 107, 190]. Moreover, astrocytes are also capable of instructing homeostatic plasticity both at the synapse and more broadly within neurons and circuits to counterbalance sustained periods of augmented activity [42, 93, 145]. Astrocytes even contribute to computation within neural networks to drive circuit and animal behavior [161, 178, 214]. Excitingly, a new study found that neuronal LTP can induce changes in astrocyte perisynaptic coverage to facilitate extended crosstalk between neighboring synapses [183]. Thus, a closer examination of how neurons and astrocytes bidirectionally interact and communicate during developmental circuit plasticity is warranted. The advent of genetic tools for visualization of astrocyte dynamics in zebrafish should provide a rich avenue for such exploration [32, 178].

Additionally, although calcium signaling in astrocyte perisynaptic processes often occurs in parallel with neuronal activity [162, 163, 167, 169], global calcium levels can act independently [166, 186]. There is recent evidence suggesting that astrocyte networks modulate autonomic control of heart rate, such that astrocyte Cx43-mediated release of ATP presumably regulates excitatory circuits in the brainstem[215]. Given the importance of local astrocytic signaling to circuit function, unraveling the importance of astrocyte-network signaling across broad brain regions is a necessary future line of research.

Finally, the rapid evolution of sequencing techniques has made the term “astrocyte” an umbrella-like category for a group of highly heterogeneous cells [13, 184, 185, 216]. Though there is some *in vivo* evidence that astrocytes can become locally specialized to provide circuit-specific support [13], this remains an open area ripe for future investigation. Future efforts should be directed at understanding how astrocytes acquire these unique expression profiles, and how this specialization guides their function within individual neurons and circuits. These types of experiments will require the development of intersectional tools that enable manipulation of specific subpopulations of astrocytes within the intact nervous system. Though challenging, the availability of single cell RNA sequencing datasets will undoubtedly speed up identification of region-specific markers that could be used for development of tools to test how functionally diverse astrocyte populations are *in vivo*, and how this diversity ensures proper neural circuit assembly and function.

## Data Availability

Data sharing is not applicable to this article as no datasets were generated or analysed during the current study.

## Abbreviations

AMPAR: α-amino-3-hydroxy-5-methyl-4-isoxazolepropionic receptors
APP: Amyloid precursor protein
ATP: Adenosine triphosphate
Chrdl1: Chordin like-1
CNS: Central nervous system
CSPG: Chondroitin sulfate proteoglycan
Cx: Connexin
E/I balance: Excitation to inhibition balance
GABA: Gamma aminobutyric acid
GJ: Gap junction
LTD: Long term depression
LTP: Long term potentiation
NMDAR: N-methyl-D-aspartate receptors
NRCAM: Neuronal cell adhesion molecule
RGC: Retinal ganglion cell
ODP: Ocular dominance plasticity
PNN: Perineuronal net
SPARC: Secreted Protein Acidic and Rich in Cysteine
TRPA1: Transient Receptor Potential Cation Channel Subfamily A Member 1
TTX: Tetrodotoxin
